# Negative parenting behaviour as a mediator of the effects of telephone-assisted self-help for parents of pharmacologically treated children with attention-deficit/hyperactivity disorder

**DOI:** 10.1007/s00787-020-01565-w

**Published:** 2020-06-02

**Authors:** Christina Dose, Christopher Hautmann, Mareike Bürger, Stephanie Schürmann, Manfred Döpfner

**Affiliations:** 1 School for Child and Adolescent Cognitive Behavior Therapy (AKiP), Faculty of Medicine and University Hospital Cologne, University of Cologne, Pohligstr. 9, 50969 Cologne, Germany; 2 Department of Child and Adolescent Psychiatry, Psychosomatics and Psychotherapy, Faculty of Medicine and University Hospital Cologne, University of Cologne, Robert-Koch-Str. 10, 50931 Cologne, Germany

**Keywords:** Attention-deficit/hyperactivity disorder, Methylphenidate, Telephone-assisted self-help, Mediation, Parenting behaviour

## Abstract

A previous randomised controlled trial demonstrated the effects of a telephone-assisted self-help (TASH) intervention for parents of pharmacologically treated children with attention-deficit/hyperactivity disorder (ADHD) on ADHD symptoms, oppositional symptoms, functional impairment, and negative parenting behaviour (per-protocol analyses). In the current study, we examined whether changes in positive and negative parenting behaviour mediated the effects on symptoms and impairment. Parents in an enhancement group (*n* = 51) participated in a 12-month TASH intervention (eight booklets plus up to 14 telephone consultations) as an adjunct to routine clinical care, whereas parents in a waitlist control group (*n* = 52) received routine clinical care only. Parents completed measures of child symptoms, child functional impairment, and parenting behaviour at baseline, at 6 months, and at 12 months. The mediating effects of parenting behaviour were examined using regression analyses. Per-protocol analyses (*n* = 74) revealed a significant indirect intervention effect on functional impairment through negative parenting behaviour at 6 months as well as indirect intervention effects on oppositional symptoms and functional impairment through negative parenting behaviour at 12 months. The indirect effect on ADHD symptoms through negative parenting behaviour at 12 months just failed to reach significance. The analyses yielded no indirect intervention effects through positive parenting behaviour. The study provides some, albeit limited, support for the importance of changes in negative parenting behaviour to achieve changes in symptoms and functional impairment during parent training. In consideration of the inconsistent results of previous studies concerning the mediating role of positive and negative parenting behaviour, further research is required to better understand the mechanisms of change during parent training, also including other possible mediators like parenting stress and parental self-efficacy.

## Introduction

Behavioural interventions, especially parent training, are widely accepted and recommended treatment options for school-age children with attention-deficit/hyperactivity disorder [1–3]. Often, parenting interventions are embedded in a multimodal therapy plan, which may additionally include teacher-focused interventions (for instance psychoeducation) and child-focused treatment (for instance psychoeducation, cognitive behavioural therapy of the child, or medication) [1]. Given the shortage of face-to-face therapy options in Germany and other European countries [4, 5], and several other treatment barriers such as a lack of time or financial resources and fear of stigmatisation [5, 6], there is a growing interest in self-help interventions which may help to overcome some of these barriers [6, 7]. Extensive research has shown that both face-to-face and self-help interventions, especially parenting interventions, are effective in the treatment of school-age children with ADHD, at least with regard to parent-rated outcomes [8–11]. However, blinded ratings of ADHD symptoms often do not yield effects of face-to-face or self-help parenting interventions [8, 11, 12].

Despite evidence to support the efficacy of parent training for the treatment of ADHD, and the widespread use of such interventions, little is known about the mechanisms by which the interventions exert their effects. Knowledge of these mechanisms might help to improve therapy outcomes [13]. The general idea of parent training is to teach behavioural modification techniques to parents, which they can use to deal with the behaviour problems of their child. This should lead to a change in parenting behaviour, which should in turn bring about an improvement in the child’s symptoms [14]. Parent training for the treatment of children with ADHD is often based on parenting interventions for the treatment of other externalising disorders, that is, oppositional defiant disorder (ODD) and conduct disorder (CD) [8]. Many of them are based on the assumption of coercive interactional cycles between parent and child, which lead to the development of behaviour problems [8]. To break these cycles, parents are trained to reinforce appropriate child behaviours, to discourage noncompliant child behaviours, and to enhance positive parent–child interactions [8].

In the field of CD and ODD or externalising behaviour disorders in general (that is, CD, ODD, and ADHD taken together), several studies have already examined a change in parenting behaviour as a putative mediator of the effects of parent training [15–23]. Besides constructs such as discipline and monitoring/supervision, studies often consider positive and negative parenting behaviours as potential mediating variables [17]. Positive parenting behaviour includes, for example, the use of praise, encouragement, effective communication, and joint play [17, 24]. Negative parenting behaviour, on the other hand, comprises, for example, verbal criticism and harshness [17]. Previous research has yielded inconclusive findings regarding the indirect effects of parent training via a change in positive and negative parenting behaviours. While some studies reported that improved positive parenting behaviour mediated the effects of parent training [19, 20], others only found evidence for negative parenting behaviour as a mediator [15, 18], and others still reported mediation effects through both positive and negative parenting behaviour [23]. The latter was also found for the mediation of the effects of a self-help intervention on conduct problems [25].

To date, however, only a small number of studies have specifically focused on mechanisms of change in the treatment of children with a diagnosis of ADHD. Chronis-Tuscano et al. reported that negative parenting behaviour, but not positive parenting behaviour, mediated the association between maternal ADHD symptoms and improvement in mother-rated child behaviour following brief behavioural parent training [26]. Booster et al. also pointed out the mediating role of reduced negative parenting behaviour. The authors found that the effects of a family-school intervention on teacher-reported homework responsibility and parent-rated homework problems were mediated by reductions in negative parenting, but again not by improvements in positive parenting [27]. In line with these findings, Haack et al. reported that negative parenting behaviour mediated the effects of a parent-focused intervention on several parent- and teacher-rated outcomes [28]. In a multicomponent intervention which also included a child-focused and a teacher-focused component, the authors additionally detected that a change in positive parenting behaviour mediated the intervention effects on parent-rated social skills [28].

In the present study, we examined parenting behaviour as a putative mediator of the effects of a behaviourally oriented telephone-assisted self-help (TASH) intervention for parents of children with ADHD, which consisted of eight self-help booklets plus 14 counselling telephone calls. The efficacy and effectiveness of this intervention have already been demonstrated in several studies in preschool-age and school-age children with externalising behaviour disorders [29–33]. Possible advantages of telephone-assisted self-help interventions comprise time and cost savings, the possibility to treat patients in case of a lack of local treatment options (or to offer them treatment while waiting for local treatment), the opportunity to reach patients who are afraid of stigmatization, as well as the opportunity to increase availability of local treatment options for more severely affected patients by successfully treating patients for whom self-help interventions are sufficient. Possible disadvantages are the complication of the diagnostic process due to the missing face-to-face contact and limited opportunities for therapeutic interventions like role plays to practise new patterns of behaviour or acting as a role model. Moreover, there are a limited number of participants who may take part in the telephone consultations. A recent study revealed that parental attributions mediated the effect of the TASH intervention on ADHD symptoms, ODD symptoms, and externalising problems in children with externalising behaviour disorders as compared to a nondirective telephone-assisted self-help intervention [34]. The latter study found no mediating effects through positive parenting behaviour, negative parenting behaviour, or parental self-efficacy [34].

Data for the current analyses were based on a previous randomised waitlist-controlled trial on the effects of the TASH intervention in a sample of school-age children with ADHD who experience functional impairment despite methylphenidate treatment. Intention-to-treat analyses yielded intervention effects on oppositional defiant disorder (ODD) symptoms (*d* = 0.43) as well as on negative parenting behaviour (*d* = 0.48) [35]. Moreover, per-protocol analyses, which included only families who had completed the intervention, additionally demonstrated effects of the TASH intervention on ADHD symptoms and functional impairment (ADHD symptoms: *d* = 0.52, ODD symptoms: *d* = 0.64, negative parenting: *d* = 0.62, and functional impairment: *d* = 0.71) [35]. The effects on ODD symptoms, functional impairment, and negative parenting behaviour persisted when controlling for a possible confounding effect of a change in medication during the intervention [35]. However, the per-protocol effect on ADHD symptoms should be interpreted with caution, as different results emerged depending on whether or not missing values for a change in medication were imputed [35].

With the current analyses, we aimed to examine whether changes in positive and negative parenting behaviour mediate the effects of the TASH intervention on ADHD symptoms, ODD symptoms, and functional impairment. We assumed that the TASH intervention would lead to higher levels of positive and lower levels of negative parenting behaviour, which, in turn, would lead to lower levels of ADHD symptoms, ODD symptoms, and functional impairment in an enhancement group compared to a waitlist control group. Although a current study on the TASH intervention in a sample of children with ADHD and/or ODD did not yield any significant mediating effects through positive or negative parenting behaviour [34], we concentrated on these variables and tested the aforementioned hypotheses as they are consistent with the theoretical assumption that the effects of parent training are mainly induced through a change in parenting behaviour. Moreover, in line with our hypotheses, another study found that a self-help intervention for children with conduct problems achieved its effects through a change in both positive and negative parenting behaviour [25]. Finally, studies exclusively focusing on face-to-face parent training for parents of children with a diagnosis of ADHD yielded support for the mediating effects of either negative parenting behaviour or both positive and negative parenting behaviour [26–28]. To our knowledge, the current study is the first study to examine the indirect intervention effects of a TASH intervention through positive and negative parenting behaviour in a sample of medically treated school-age children with ADHD.

## Methods

The study was registered at ClinicalTrials.gov (identifier: NCT01660425; URL: https://clinicaltrials.gov/ct2/show/NCT01660425).

### Design

The study design was a randomised parallel-group trial with two treatment arms. Parents were randomised to either an enhancement group (EG) or a waitlist control group (WCG) using computerised block randomisation [35]. Parents in the EG (*n* = 51) participated in a 12-month TASH intervention as an adjunct to routine clinical care, while parents in the WCG (*n* = 52) received routine clinical care only. At the beginning of the study, all children were receiving treatment with methylphenidate. If indicated, the attending physician continued and monitored this treatment [35].

### Participants

Parents were eligible for the study if they met the following inclusion criteria (see also [35]): their child was aged 6–12 years, attended school, had already been diagnosed with ADHD by a paediatrician or psychiatrist, and was already being treated with methylphenidate. Furthermore, the methylphenidate dose had been stable for at least 2 months, with no planned change of active substance or dose. The child had to show functional impairment as indicated by the Weiss Functional Impairment Rating Scale-Parent Report (WFIRS-P) [36], as this was the primary outcome of the main study. Moreover, the parents had to be motivated to participate and needed to have sufficient knowledge of the German language to work with the written self-help materials provided in the study. If the child was participating in a behaviour therapy, the parents were still eligible for inclusion provided that the parents were not regularly involved in the therapy. The recruitment period lasted from May 2012 to November 2013. For recruitment purposes, we sent study information by post to approximately 3600 registered child psychiatrists, paediatricians, child guidance offices, and social-psychiatric service centres in Germany. Moreover, we promoted the study on the Internet [35].

### Intervention: TASH

EG parents participated in a 12-month behavioural TASH intervention, which consisted of eight self-help booklets on externalising behaviour disorders and parenting strategies [37] and up to 14 accompanying telephone consultations of about 30 min each. We provided the parents with the eight booklets and ten telephone consultations during the first 6 months of the intervention (intensive phase). In the subsequent booster phase, the parents received four more telephone consultations [35]. The contents of the booklets were as follows: (1) definition of individual problem behaviour and information on coercive parent–child interactions, (2) psychoeducation, (3) encouragement of positive parent–child interactions, (4) implementation of family rules and effective demands, (5) appropriate positive and negative consequences of obeying or breaking rules, (6) promoting strengths of the child and advice for some specific problem situations (e.g., use of media, resolving conflicts with peers), (7) developing everyday structures and stress reduction for parents, and (8) reward systems.

Treatment integrity was ensured by audio recordings of the telephone consultations, which were supervised regularly. In addition, the counsellors completed a checklist on treatment integrity after each session. According to the average ratings on this checklist, 90% of the topics of the booklets were discussed and 5% were “partially” discussed (see also [35]). Moreover, the counsellors asked the parents some questions on implementation fidelity at the beginning of each session. In 98.7% of the consultations, parents indicated that they had read the booklet that was planned to be subject of the consultation. In 92.4% of cases, parents stated that they had well understood the contents of the booklets, while in 7.6% of cases, they told that they had partly understood the contents. Moreover, in 74.0% of cases, parents indicated that they found the advice given in the booklets helpful; in 22.9% of cases, they rated the advice as partly helpful; and in 3.2% as not helpful. For 58.5% of the booklets contents, parents expressed that they were frequently able to use the techniques they were trained in; for 38.3%, they stated that they used the advice partly or seldom; and for 3.2% of the advice given they indicated that they never used it.

### Measures

The participating parents completed measures of ADHD symptoms, oppositional defiant disorder (ODD) symptoms, functional impairment, and parenting behaviour at baseline, at 6 months and at 12 months (post-assessment). The questionnaires were sent and returned by post. If possible, we collected missing data by telephone [35].

Parents rated child ADHD and ODD symptoms on the Symptom Checklist for Attention-Deficit/Hyperactivity Disorder (German: "Fremdbeurteilungsbogen für Aufmerksamkeitsdefizit-/Hyperaktivitätsstörungen", FBB-ADHS) [38] and on the *ODD* subscale of the Symptom Checklist for Oppositional Defiant Disorder and Conduct Disorder (German: "Fremdbeurteilungsbogen für Störungen des Sozialverhaltens", FBB-SSV) [38]. The FBB-ADHS comprises 20 items which assess ADHD symptoms according to ICD-10 and DSM-IV. The ODD subscale of the FBB-SSV consists of nine items capturing symptoms of ODD as defined by the classification systems. All items are rated on a 4-point Likert-type scale ranging from 0 to 3, with higher scores indicating greater symptom severity. Scale scores are computed by averaging the associated item scores. The scales have demonstrated internal consistency (*α* > 0.80) for all subscales and the total scores as well as factorial validity [38–41].

To assess functional impairment, we applied a modified, German-language version of the WFIRS-P [36]. The original WFIRS-P comprises 50 items on functional impairment in different domains: (a) family, (b) learning and school, (c) life skills, (d) child’s self-concept, (e) social activities, and (f) risky activities. We excluded the scale assessing risky activities as, in our opinion, this scale captures externalising symptoms rather than impairment [35]. The parents rated the remaining 40 items on a 4-point Likert-type scale ranging from 0 to 3, with higher scores indicating more severe impairment. The WFIRS-P has demonstrated good internal consistency (*α* > 0.70), factorial validity, test–retest reliability, divergent and convergent validity, and responsiveness to change [36, 42–45].

In addition, the parents rated their positive and negative parenting behaviour on the Positive and Negative Parenting Questionnaire (German: “Fragebogen zum Positiven und Negativen Erziehungsverhalten”, FPNE) [46]. This questionnaire is based on the revised version of the Management of Children’s Behavior Scale [47] and on the Parent Practises Scale [48], but also encompasses some new items on specific aspects of behavioural parent training (e.g., handling of family rules). The FPNE consists of 38 items which are rated on a 4-point Likert-type scale, with higher scores indicating more positive or more negative parenting behaviour, respectively. Two scales capturing positive and negative parenting may by derived by averaging the associated item scores. Both questionnaires which the FPNE is based on and the FPNE itself have demonstrated sound psychometric properties. For the Management of Children’s Behavior Scale, psychometric analyses revealed internal consistency, sensitivity to change, as well as concurrent and predictive validity [47]. Also, internal consistency and construct validity have been shown for the Parent Practises Scale [48]. The two scales of the FPNE have demonstrated satisfactory internal consistency [46].

### Missing values and statistical analyses

The analyses were performed using the Statistical Package for the Social Sciences, SPSS version 24 (IBM Corporation, Armonk, NY, USA). We performed per-protocol analyses; that is, we considered all families who had completed the intervention (*n* = 74). We only regarded families who were actually trained in parenting techniques, and, thus, were more likely to change their parenting behaviour, as we assumed that this approach would be of greater practical relevance for the mediation analyses than intention-to-treat analyses (which would also take into account families with early discontinuation). EG families were included in the per-protocol sample if they had received all eight booklets, had made use of at least nine telephone consultations, and had completed the post-assessment (*n* = 33). WCG families were selected for this sample if they had completed the post-assessment (*n* = 41; see also [35]).

There was one missing value for an item of the ODD scale in the EG both at baseline and at post-assessment. The ODD scale score for the family concerned was determined by averaging the available item scores. Moreover, three families did not return the questionnaires at the 6-month assessment point. For these families, we imputed missing values for positive and negative parenting behaviour using the expectation maximisation (EM) method separately for the two variables, considering baseline data and available data at 6 months as predictors.

To examine whether positive and negative parenting behaviours mediate the effects of the TASH intervention on ADHD symptoms, ODD symptoms, and functional impairment, we performed mediation analyses using the SPSS macro PROCESS designed by Hayes [49]. PROCESS uses ordinary least-squares (OLS) regression to estimate the model parameters.

In a simple mediation model, an independent variable *X* indirectly affects a dependent variable *Y* through a mediating variable *M* [49, 50]. The total effect of *X* on *Y* (*c*) is divided into a direct (*c′*) and an indirect effect (*ab*) through the mediating variable [50]. The indirect effect (*ab*) is the product of the effect of *X* on the mediating variable *M* (*a*) and the effect of the mediating variable on *Y* (*b*) [50]. The direct effect *c′* is the effect of *X* on *Y* when controlling for the mediator [49].

When several variables are examined as possible mediating variables, it is recommended to consider them together in a multiple mediation model [49–51]. In this model, the so-called specific indirect effect through one of the mediating variables is the product of *M* regressed on *X* and of *Y* regressed on *M*, controlling for the influence of the other mediators in the model [49]. The total indirect effect is the sum of the specific indirect effects, but is of lesser interest. As in the simple mediation model, the indirect effects and the direct effect add up to the total effect [49].

**Fig. 1:**
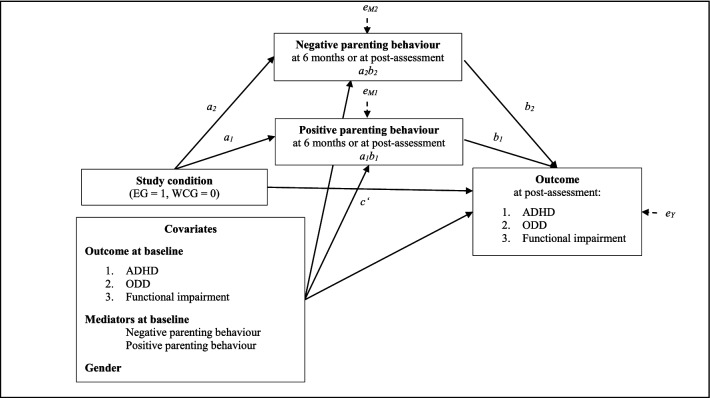
Multiple mediator model for the mediation of effects of a telephone-assisted self-help intervention. Six different models were considered: one with ADHD symptoms, one with ODD symptoms and one with functional impairment serving as dependent variable and baseline covariate, each with positive and negative parenting behaviour at 6 months as mediators; and one with ADHD symptoms, one with ODD symptoms and one with functional impairment serving as dependent variable and baseline covariate, each with positive and negative parenting behaviour at 12 months as mediators. *EG* enhancement group, *WCG* waitlist control group, *ADHD* attention-deficit/hyperactivity disorder, *ODD* oppositional defiant disorder

In total, we tested six different models (see Fig. 1). We considered ADHD symptoms, ODD symptoms, and functional impairment as dependent variables and study condition (EG vs. WCG, coded as 1 vs. 0) as independent variable. In three models, we regarded positive and negative parenting behaviour at 6 months as mediating variables; in the other three models, positive parenting behaviour and negative parenting behaviour at 12 months were the mediating variables. In all cases, we tested the hypothetical model that the TASH intervention would lead to a change in positive and negative parenting behaviour, which would then lead to a change in ADHD symptoms, ODD symptoms, or functional impairment. We examined both models with parenting behaviours at 6 months and parenting behaviours at 12 months as mediators as we took into consideration that the 6-month period may not have been sufficiently long to achieve profound changes in parenting behaviour and, accordingly, detect a mediating effect (as the intensive part of the intervention lasted 6 months and we assumed that the parents would need some more time to actually implement the newly acquired parenting techniques).

Following the recommendation of Hayes, we did not use baseline-post-assessment difference scores for the mediators and the dependent variables: These tend to be correlated with earlier measurements and the interpretation of the according results might be complicated due to artefacts such as regression to the mean or ceiling and floor effects [49]. Instead, we used the post-assessment values as dependent variables and included the baseline values of the respective outcome variable and the mediators as covariates in each model to control for possible baseline group differences [49, 52]. Moreover, we included gender as a further covariate in the analyses, as we found a significant group difference for this variable (see “Results” section).

There is nowadays general consensus that a statistically significant total effect of the independent variable on the dependent variable is not a prerequisite for mediation analyses to be performed [49, 53]. Therefore, we also interpreted the indirect effects in models for which the analyses did not yield a total effect. Moreover, we focus on the significance of the product *ab* when interpreting indirect effects instead of considering the significance of the paths *a* and *b* that define this indirect effect [49]. Following Hayes, it is important to consider the signs of *a* and *b*, as they determine the sign of the indirect effect *ab* and because they are relevant for its interpretation [49]. However, the significance of the product *ab* is sufficient to claim the existence of an indirect effect [49]. This is in contrast to the causal steps approach [54], which was formerly often used to approach mediation and which actually assumes a significant total intervention effect of the study condition on the outcome, a significant association between study condition and the mediator, and a significant association between the mediator and the outcome for an indirect intervention effect to be established. However, Hayes [49] presents several arguments why this approach is regarded critically in current methodology literature. Inter alia, he states that one of the main problems of this approach is that it does neither directly quantify the indirect effect nor test its significance, but infers logically from a set of hypotheses about other effects that there has to be an indirect effect. The proper estimate of the indirect effect is the product of the paths *a* and *b*; thus, inference should be based on this product [49].

As recommended by Hayes, we report unstandardised regression coefficients [49]. We computed percentile bootstrap confidence intervals using 10,000 bootstrap samples. The random number generator for bootstrapping was seeded with the value 54,321 to allow for replication of the bootstrap samples. Effects were considered as significant if the 95% confidence interval did not include zero. To gain an impression of the effect sizes of the specific indirect, direct, and total effects, we considered partially standardised effects, which express the effects relative to the standard deviation of the dependent variable [49]. To examine the goodness of fit of our proposed models, we considered the proportion of variance explained by different parts of the models (that is, the mediating variables regressed on study condition and the covariates, and the dependent variable regressed on study condition, the mediating variables and the covariates), expressed by *R*^2^ [49].

We hypothesised that changes in parenting behaviour would lead to changes in ADHD symptoms, ODD symptoms and functional impairment. To investigate this assumption adequately, the assessment of the mediators should precede the assessment of the outcomes [49]. This holds for the models with parenting behaviours assessed at 6 months as mediators. However, in the models considering positive and negative parenting behaviour at 12 months as mediating variables, we assessed our mediating variables and outcomes at the same time point. Thus, in these models, we cannot rule out the possibility that a reverse order of the variables in our models could actually be closer to reality, that is, that the TASH intervention leads to a change in symptoms and functional impairment, in turn affecting parenting behaviour. To examine such an opposite direction of causal flow, we analysed two alternative models. In both models, study condition was considered as independent variable and ADHD symptoms, ODD symptoms, and functional impairment were treated as mediators. One of the models included positive parenting behaviour as dependent variable, while the other considered negative parenting behaviour as dependent variable.

## Results

### Sample characteristics

Detailed information on the participant flow and the sample characteristics is reported in the publication of the main study [35]. In total, 18 families were considered as “non-completing families” and, thus, were not included in the per-protocol sample considered in this study. Five of these families dropped out after having received the first booklet (before the first telephone consultation), four families discontinued the intervention after the first consultation, one family dropped out after the forth consultation, one family after the fifth, and three families after the sixth session. Four further families were not considered for the per-protocol sample although they had participated in the minimum number of nine consultations because they did not return the final measurement at 12 months. Parents who completed the intervention participated in an average number of 13.64 (SD = 1.10; range 9–14) telephone consultations.

The mean age of the EG children in the per-protocol sample was 9.86 years (SD = 1.47); the mean age of the WCG children was 9.71 years (SD = 1.77). A Chi-square test revealed a significant group difference in gender (EG: 70%, WCG: 90%; *χ*^2^ = 5.03, *df* = 1, *p* = 0.04) and an independent-samples *t* test yielded a significant group difference in positive parenting behaviour at baseline, with the EG families demonstrating more positive parenting behaviour (*t* = 2.73, *df* = 72, *p* < 0.01). No significant group differences at baseline emerged with regard to age, ADHD symptoms, ODD symptoms, functional impairment, or negative parenting behaviour.

EG families who completed the intervention and families with early discontinuation differed significantly on some variables: In completing families, parents had more educational years [completers: *M* = 12.74, SD = 2.66; noncompleters: *M* = 10.00, SD = 3.22; *t* = 3.22, *df* = 47, *p* < 0.01 (two missing values in the completing families)], and there were fewer siblings living in the same household as the participating child (completers: *M* = 0.94, SD = 0.66; noncompleters: *M* = 1.50, SD = 1.04; *t* = − 2.35, *df* = 49, *p* = 0.02). Moreover, completers demonstrated more functional impairment on the WFIRS-P subscale on life skills (completers: *M* = 1.18, SD = 0.55; noncompleters: *M* = 0.83, SD = 0.45; *t* = 2.29, *df* = 49, *p* = 0.03), higher scores on a scale measuring social competences (completers: *M* = 1.58, SD = 0.48; noncompleters: *M* = 1.17, SD = 0.43; *t* = 3.00, *df* = 49, *p* < 0.01), and less negative parenting behaviour at baseline (completers: *M* = 1.24, SD = 0.30; noncompleters: *M* = 1.47, SD = 0.35; *t* = − 2.42, *df* = 49, *p* = 0.02). In the WCG, there were no differences between completing families and families with early discontinuation at baseline.

### Mediation of intervention effects of TASH on symptoms and functional impairment through parenting behaviour

The total effect of the TASH intervention on ODD symptoms reached significance, while the intervention effects on ADHD symptoms and functional impairment did not (but were very close to significance; see Tables 1 and 2). Considering the models with positive and negative parenting behaviour at 6 months as mediators, there was a significant negative association between positive parenting behaviour and the outcome (ADHD, ODD, or functional impairment at 12 months) as well as a significant negative association between study condition and negative parenting behaviour in all three models. Moreover, the analyses yielded a significant positive association between negative parenting behaviour and functional impairment. This reveals that negative parenting behaviour showed a stronger reduction in the EG which, in turn, was associated with functional impairment. Also, we found a significant specific indirect intervention effect on functional impairment through negative parenting behaviour. The partially standardised specific indirect effect through negative parenting behaviour was − 0.210 in the respective model. That is, two given cases in different study conditions differ in functional impairment by about one-fifth of a standard deviation as a result of the indirect intervention effect through negative parenting behaviour. All other indirect effects through positive or negative parenting behaviour as well as the direct effects of study condition on the respective outcome were non-significant in the models considering parenting behaviours at 6 months as mediators (see Table 1). In all models, study condition and the covariates taken together explained between 53 and 71% of the variance in the mediators. Moreover, study condition, the covariates, and the mediators accounted for more than 40% of the variance in ADHD symptoms, ODD symptoms, and functional impairment at post-assessment (ADHD: *R*^2^ = 0.43, ODD: *R*^2^ = 0.42, functional impairment: *R*^2^ = 0.42).

**Table 1:** Unstandardised regression coefficients, bootstrap confidence intervals, and model information for the multiple mediator model for the mediation of the effects of a telephone-assisted self-help intervention depicted in Fig. 1 (mediators: positive and negative parenting behaviour at 6 months)

	Outcome											
	ADHD				ODD				FI			
	Coeff.	Bootstrap SE	95% Bootstrap CI	Partially stand. effect	Coeff.	Bootstrap SE	95% Bootstrap CI	Partially stand. effect	Coeff.	Bootstrap SE	95% Bootstrap CI	Partially stand. effect
*a*_1_	0.035	0.044	− 0.049; 0.121		0.039	0.044	− 0.044; 0.126		0.034	0.044	− 0.049; 0.121	
*b*_1_	− 0.640^a^	0.357	− 1.434; − 0.038		− 0.931^a^	0.356	− 1.721; − 0.321		− 0.531^a^	0.226	− 1.014; − 0.128	
*a*_1_*b*_1_	− 0.022	0.039	− 0.127; 0.023	− 0.035	− 0.036	0.048	− 0.153; 0.036	− 0.051	− 0.018	0.028	− 0.090; 0.020	− 0.041
*a*_2_	− 0.160^a^	0.052	− 0.267; − 0.061		− 0.168^a^	0.053	− 0.274; − 0.066		− 0.168^a^	− 0.053	− 0.277; − 0.068	
*b*_2_	0.269	0.287	− 0.343; 0.792		0.382	0.349	− 0.308; 1.052		0.556^a^	0.219	0.099; 0.959	
*a*_2_*b*_2_	− 0.043	0.050	− 0.147; 0.057	− 0.068	− 0.064	0.067	− 0.218; 0.048	− 0.089	− 0.093^a^	0.047	− 0.196; − 0.013	− 0.210
*c′*	− 0.198	0.146	− 0.468; 0.107	− 0.311	− 0.292	0.167	− 0.601; 0.052	− 0.405	− 0.078	0.109	− 0.286; 0.140	− 0.176
*c*	− 0.264	0.133	− 0.528; 0.001	− 0.413	− 0.392^a^	0.156	− 0.703; − 0.081	− 0.544	− 0.190	0.100	− 0.390; 0.010	− 0.427

Sample size *n* = 74

*a*_*1*_ study condition → positive parenting behaviour (at 6 months), *b*_*1*_ positive parenting behaviour (at 6 months) → outcome (at 12 months), *a*_*1*_*b*_*1*_ indirect effect of study condition on outcome through positive parenting behaviour, *a*_*2*_ study condition → negative parenting behaviour (at 6 months), *b*_*2*_ negative parenting behaviour (at months) → outcome (at 12 months), *a*_*2*_*b*_*2*_ indirect effect of study condition on outcome through negative parenting behaviour, *c′* direct effect of study condition on outcome, *c* total effect of study condition on outcome, *ADHD* attention-deficit/hyperactivity disorder, *ODD* oppositional defiant disorder, *FI* functional impairment, *Coeff.* unstandardised regression coefficient, *SE* standard error, *CI* confidence interval

^a^Significant coefficient (95% CI). The values of the mediators and the outcome variables at baseline and gender were included as covariates in the model; to improve the clarity of the table, the associations of these variables with the other variables in the model are not displayed. The standard errors and confidence intervals for the total effects were determined without the use of bootstrap samples

**Table 2:** Unstandardised regression coefficients, bootstrap confidence intervals, and model information for the multiple mediator model for the mediation of the effects of a telephone-assisted self-help intervention depicted in Fig. 1 (mediators: positive and negative parenting behaviour at post-assessment)

	Outcome											
	ADHD				ODD				FI			
	Coeff.	Bootstrap SE	95% Bootstrap CI	Partially stand. effect	Coeff.	Bootstrap SE	95% Bootstrap CI	Partially stand. effect	Coeff.	Bootstrap SE	95% Bootstrap CI	Partially stand. effect
*a*_1_	0.094	0.070	− 0.045; 0.230		0.099	0.069	− 0.039; 0.236		0.094	0.070	− 0.044; 0.228	
*b*_1_	− 0.782^a^	0.252	− 1.322; − 0.330		− 0.904^a^	0.250	− 1.417; − 0.436		− 0.610^a^	0.134	− 0.886; − 0.359	
*a*_1_*b*_1_	− 0.073	0.064	− 0.218; 0.031	− 0.115	− 0.089	0.072	− 0.250; 0.031	− 0.124	− 0.058	0.048	− 0.163; 0.023	− 0.129
*a*_2_	− 0.144^a^	0.063	− 0.263; − 0.016		− 0.158^a^	0.065	− 0.280; − 0.024		− 0.158^a^	0.066	− 0.282; − 0.022	
*b*_2_	0.452^a^	0.203	0.048; 0.847		0.825^a^	0.259	0.314; 1.340		0.525^a^	0.152	0.233; 0.831	
*a*_2_*b*_2_	− 0.065	0.043	− 0.166; 0.0003	− 0.102	− 0.130^a^	0.077	− 0.310; − 0.013	− 0.181	− 0.083^a^	0.043	− 0.176; − 0.010	− 0.186
*c′*	− 0.125	0.131	− 0.375; 0.141	− 0.197	− 0.173	0.137	− 0.435; 0.105	− 0.240	− 0.050	0.093	− 0.228; 0.135	− 0.112
*c*	− 0.264	0.133	− 0.528; 0.001	− 0.413	− 0.392^a^	0.156	− 0.703; − 0.081	− 0.544	− 0.190	0.100	− 0.390; 0.010	− 0.427

Sample size *n* = 74

*a*_*1*_ study condition → positive parenting behaviour (post-assessment), *b*_*1*_ positive parenting behaviour (post-assessment) → outcome, *a*_*1*_*b*_*1*_ indirect effect of study condition on outcome through positive parenting behaviour (post-assessment), *a*_*2*_ study condition → negative parenting behaviour (post-assessment), *b*_*2*_ negative parenting behaviour (post-assessment) → outcome, *a*_*2*_*b*_*2*_ indirect effect of study condition on outcome through negative parenting behaviour (post-assessment), *c′* direct effect of study condition on outcome, *c* total effect of study condition on outcome, *ADHD* attention-deficit/hyperactivity disorder, *ODD* oppositional defiant disorder, *FI* functional impairment, *Coeff.* unstandardised regression coefficient, *SE* standard error, *CI* confidence interval

^a^Significant coefficient (95% CI). The values of the mediators and the outcome variables at baseline and gender were included as covariates in the model; to improve the clarity of the table, the associations of these variables with the other variables in the model are not displayed. The standard errors and confidence intervals for the total effects were determined without the use of bootstrap samples

With regard to the models including parenting behaviours at 12 months as mediators, all three models yielded a negative association between study condition and negative parenting behaviour as well as a positive association between negative parenting behaviour and the respective outcome (see Table 2). In the models considering ODD symptoms and functional impairment as outcomes, the specific indirect effect of the TASH intervention through negative parenting behaviour became significant. The partially standardised specific indirect effects through negative parenting behaviour were − 0.181 in the ODD model and − 0.186 in the functional impairment model. In the model with ADHD symptoms as outcome, this specific indirect effect narrowly missed significance (partially standardised specific indirect effect through negative parenting behaviour: − 0.102; see Table 2). In all models, the specific indirect intervention effect through positive parenting behaviour and the direct effect of study condition on the respective outcome were non-significant. In all models, study condition and the covariates taken together explained a substantial proportion of variance in the mediators (range of *R*^2^: 0.47–0.55). Similarly, study condition, the covariates, and the mediators accounted for over half of the variance in the outcome variables (ADHD: *R*^2^ = 0.54, ODD: *R*^2^ = 0.57, functional impairment: *R*^2^ = 0.55).

### Mediation of intervention effects on parenting behaviour through symptoms and functional impairment

To evaluate whether the opposite direction of causal flow in the models including parenting behaviours at 12 months may be closer to reality (that is, that the intervention leads to changes in ADHD symptoms, ODD symptoms, and functional impairment, which then lead to changes in parenting behaviour), we examined two alternative models. These models included study condition as independent variable and ADHD symptoms, ODD symptoms, and functional impairment as mediators. In one model, positive parenting behaviour was considered as dependent variable, and in the other, negative parenting behaviour was considered as dependent variable.

None of the indirect effects became significant in the model considering positive parenting behaviour as dependent variable (see Table 3). Moreover, the total effect and the direct effect of study condition on positive parenting behaviour were non-significant. For the model regarding negative parenting behaviour as dependent variable, the analysis yielded a significant total effect. Moreover, there was a significant negative association between study condition and ODD symptoms, a significant positive association between ODD symptoms and negative parenting behaviour, and a significant negative specific indirect effect of study condition on negative parenting behaviour through ODD symptoms (see Table 3).

**Table 3:** Model information for the mediator models for the mediation of the effects of a telephone-assisted self-help intervention on parenting behaviour at post-assessment through symptoms and impairment

	Outcome							
	Positive parenting				Negative parenting			
	Coeff	Bootstrap SE	95% Bootstrap CI	Partially stand. effect	Coeff	Bootstrap SE	95% Bootstrap CI	Partially stand. effect
*a*_*1*_	− 0.211	0.137	− 0.476; 0.064		− 0.247	0.129	− 0.496; 0.017	
*b*_*1*_	− 0.115	0.071	− 0.254; 0.029		0.003	0.065	− 0.121; 0.133	
*a*_*1*_*b*_*1*_	0.024	0.024	− 0.013; 0.080	0.060	− 0.0008	0.018	− 0.038; 0.038	− 0.002
*a*_*2*_	− 0.339^a^	0.165	− 0.681; − 0.031		− 0.427^a^	0.156	− 0.743; − 0.130	
*b*_*2*_	− 0.105	0.066	− 0.226; 0.033		0.160^a^	0.067	0.030; 0.292	
*a*_*2*_*b*_*2*_	0.036	0.031	− 0.012; 0.108	0.088	− 0.069^a^	0.039	− 0.158; − 0.007	− 0.198
*a*_*3*_	− 0.167	0.104	− 0.376; 0.034		− 0.237^a^	0.104	− 0.446; − 0.038	
*b*_*3*_	− 0.223^a^	0.102	− 0.428; − 0.030		0.140	0.093	− 0.024; 0.344	
*a*_*3*_*b*_*3*_	0.037	0.036	− 0.007; 0.127	0.092	− 0.033	0.033	− 0.118; 0.005	− 0.096
*c ‘*	− 0.011	0.057	− 0.127; 0.098	− 0.026	− 0.070	0.060	− 0.182; 0.054	− 0.201
*c*	0.086	0.075	− 0.062; 0.235	0.214	− 0.172^a^	0.066	− 0.303; − 0.040	− 0.496

Sample size *n* = 74

*a*_*1*_ study condition → symptoms of attention-deficit/hyperactivity disorder (post-assessment), *b*_*1*_ symptoms of attention-deficit/hyperactivity disorder (post-assessment) → outcome, *a*_*1*_*b*_*1*_ indirect effect of study condition on outcome through symptoms of attention-deficit/hyperactivity disorder, *a*_*2*_ study condition → symptoms of oppositional defiant disorder (post-assessment), *b*_*2*_ symptoms of oppositional defiant disorder (post-assessment) → outcome, *a*_*2*_*b*_*2*_ indirect effect of study condition on outcome through symptoms of oppositional defiant disorder (post-assessment), *a*_*3*_ study condition → functional impairment (post-assessment), *b*_*3*_ functional impairment (post-assessment) → outcome, *a*_*3*_*b*_*3*_ indirect effect of study condition on outcome through functional impairment, *c′* direct effect of study condition on outcome, *c* total effect of study condition on outcome, *Coeff.* unstandardised regression coefficient, *SE* standard error, *CI* confidence interval

^a^Significant coefficient (95% CI). The values of the mediators and the outcome variables at baseline and gender were included as covariates in the model; to improve the clarity of the table, the associations of these variables with the other variables in the model are not displayed. The standard errors and confidence intervals for the total effects were determined without the use of bootstrap samples

## Discussion

This study examined changes in positive and negative parenting behaviour as mediators of the effects of a telephone-assisted self-help intervention in a sample of school-age children with ADHD and residual functional impairment despite methylphenidate treatment. When controlling for baseline symptoms or functional impairment, respectively, and for baseline positive and negative parenting behaviour and gender, per-protocol analyses yielded a significant total effect of the intervention on ODD symptoms, but not on ADHD symptoms or functional impairment. However, as the existence of a significant total effect is not a prerequisite for the detection of indirect effects, our results provide at least some support for the assumption that negative parenting behaviour acts as a mediator of the effects of the TASH intervention. In our analyses, negative parenting behaviour at 6 months mediated the intervention effect on functional impairment at 12 months, while negative parenting behaviour at 12 months mediated the influence of the intervention on both ODD symptoms and functional impairment at 12 months. In other words, the TASH intervention was associated with a lower level of negative parenting behaviour, which was, in turn, associated with lower levels of ODD symptoms and functional impairment. In the model considering ADHD symptoms as outcome, the mediating effect of negative parenting behaviour at 12 months just failed to reach significance. As our theoretical model assumes that a change in parenting behaviour precedes changes in symptoms and functional impairment, the results of the analyses including parenting behaviours at 6 months might question the mediating role of parenting behaviour for a change in ODD (and possibly ADHD) symptoms. On the other hand, it might be possible that the 6-month assessment point was set too early to detect a mediating effect. At 6 months, the parents had just completed the intensive phase of the TASH intervention; conceivably, they needed some time afterwards to actually implement the parenting techniques which they had learned about (and to achieve changes in symptoms and impairment through these techniques). Of note, some missing values had to be imputed for these analyses, constituting a possible source of bias.

The analyses did not yield any significant indirect intervention effects through positive parenting behaviour. Although the indirect effects through negative parenting behaviour were not significant in all of our models, the tendency for negative parenting behaviour to act as a mediating variable is in line with the results of several previous studies, suggesting that a change in negative parenting behaviour, but not in positive parenting behaviour, mediates the effects of parent training [18, 27, 28]. While there are also some studies which only support the assumption of positive parenting behaviour as a mediator of parent training [19, 20], Forehand et al. concluded in a systematic review that more studies pointed to indirect effects of parent training through a composite measure of parenting and discipline than through positive parenting behaviour alone [17]. However, the authors emphasise that positive parenting was part of all composite measures which they examined and, thus, may be important in combination with other parenting behaviours, for example, by constituting a basis for other parenting skills [17].

The fact that we found a significant indirect intervention effect on functional impairment (and possibly ODD symptoms) through negative parenting behaviour, but not on ADHD symptoms might indicate that a change in parenting practises is more relevant for improving functional outcomes and comorbid oppositional symptoms than for reducing ADHD core symptoms. This might be due to the fact that the interventions that aim to change negative parenting generally target problems arising secondarily from ADHD symptoms rather than the core symptoms themselves (i.e., they often target aspects of functional impairment). Moreover, comparable to many other behavioural parenting interventions for the treatment of ADHD, the TASH intervention aims to change coercive interactional cycles that are supposed to lead to the development of behaviour problems [8]. This idea originates from interventions for the treatment of oppositional and conduct problems [8], which might explain why the analyses hint on a possible indirect intervention effect on ODD symptoms, but not on ADHD symptoms, through negative parenting behaviour. There might be other variables which mediate the intervention effects on ADHD symptoms, but which have not been examined in the current study (see below). On the other hand, the indirect intervention effect on ADHD symptoms through negative parenting behaviour at 12 months just failed to reach significance, which might also be due to power issues.

Interestingly, when we reversed the order of the mediators and outcome variables in our models including parenting behaviours at 12 months, we detected a significant indirect effect of the intervention on negative parenting behaviour through a change in ODD symptoms. Thus, our analyses do not clearly indicate whether a change in negative parenting behaviour precedes a change in symptoms, or whether a change in ODD symptoms leads to a change in negative parenting behaviour. It is also possible that there is a reciprocal interplay between these variables and that a particular temporal order in which the variables change cannot be established. For example, Gershoff et al. found in a longitudinal study that early maternal use of spanking was associated with increased child externalising problems and that early child externalising problems were linked to later spanking [55]. This question of directionality requires further research.

Although our results generally hint at the importance of negative parenting behaviour as a mediating variable for the effects of parent training, it is possible that there are other aspects of parenting which also mediate the intervention effects, for example prompting, scaffolding, or monitoring [17, 28]. Moreover, there may be further putative mediators besides parenting behaviour. For example, Katzmann et al. reported that a change in parental attributions mediated the change in the TASH intervention examined in the present study as compared to a nondirective self-help intervention [34]. Heath et al. did not specifically examine a mediation model, but found that parents experienced lower levels of parenting stress and improved self-efficacy following behavioural parent training [56]. Moreover, in the same study, a clinically significant reduction in ADHD symptoms was associated with lower parental stress and higher parental self-efficacy [56]. Although the latter analysis did not allow for any causal conclusions, one might hypothesise that parental stress and self-efficacy serve as potential mediating variables. On the other hand, Gardner et al. found no support for a mediating role of parental mood or sense of competence [19], and Hanisch et al. were unable to establish parental self-efficacy as a mediating variable [23]. The precise contribution of such further putative mediators to the mediation of the effects of parenting interventions remains to be examined, especially in the field of self-help interventions.

Notably, we detected neither a significant per-protocol intervention effect on ADHD symptoms nor a significant per-protocol intervention effect on functional impairment (although these effects were close to significance), in contrast to the publication of the main results of the study [35]. This discrepancy can be explained by the fact that we included more covariates in the present analyses, the publication of the main results only controlled for baseline symptoms or baseline functional impairment and gender [35]. In the current analyses, we additionally controlled for baseline levels of positive and negative parenting behaviours to take into account baseline group differences regarding these variables. Following Hayes [49], in most cases, it is reasonable to consider the influence of the covariates on both the mediators and the outcome in a mediation model. The fact that we controlled for baseline parenting behaviours in the outcomes might explain the different total effects described here and in the publication of the main study [35].

Several limitations of the present study should be mentioned. First, and perhaps most importantly, we assessed the mediators and outcome variables in the analyses including parenting behaviours at 12 months as mediators at the same time point. Thus, due to the design of the study, it cannot be verified that changes in the mediators preceded changes in the outcomes. However, some authors argue that if one’s causal claims arise from a solid theory, it is still feasible to conduct mediation analyses despite limitations of the data, as long as the results are interpreted with caution and in consideration of the limitations of the data [49]. In our study, we found at least partial support both for models considering parenting behaviour as a mediator and symptoms or functional impairment as outcomes and for models displaying the reverse order of the variables. Thus, although we theoretically assumed that the TASH intervention would lead to a change in parenting behaviour, which would, in turn, lead to a change in symptoms, our results do not clearly strengthen this interpretation. Moreover, analyses considering parenting behaviours at 6 months as mediators only yielded support for the mediating role of negative parenting behaviour with regard to functional impairment (although the results of these analyses have to be interpreted with caution due to the imputation of missing values). Second, as most of the participating parents already showed high levels of positive parenting behaviour at baseline, there was limited room for improvement on this variable. This might have limited the possibility to find a mediating effect through positive parenting behaviour. On the other hand, it might be a weakness of the intervention that it is not able to evoke changes in positive parenting behaviour. Third, the participating parents rated all outcome variables and mediators. There is some ongoing discussion regarding the reliability of parent ratings as opposed to blinded ratings [8], and it cannot be ruled out that aspects such as social desirability or effort justification might have influenced the parents’ ratings. Fourth, we only considered the mediating effects of positive and negative parenting behaviour. As argued above, there might be other putative mediators of the effects of parent training which might be worth considering. Fifth, the differences between the EG families who completed the intervention and those with early discontinuation regarding the number of educational years of the parent, the number of siblings living in the same household, child prosocial behaviour, and negative parenting behaviour at baseline might limit the generalizability of the results and additionally provide hints on obstacles which might prevent families from completing the intervention. One might hypothesise that the TASH intervention is easier to implement in families with a higher educational level and fewer stresses. Sixth, as the analyses presented in this article were secondary analyses, the sample size for the study was determined under the assumption of the detection of moderate effects in the main analyses, which were univariate analyses of covariance [35]. There were no separate power analyses for the mediation analyses presented here. However, according to the results of a simulation study by Fritz and MacKinnon [57], the sample size available for these analyses might have been too small for some of the indirect effects to become significant. Finally, treatment integrity was only rated by the counsellors, and aspects of implementation fidelity were only rated by the participating parents. There were no ratings of these aspects by independent observers, allowing for potential bias.

To conclude, in line with the results from the previous literature, the results of our study provide some, albeit limited, support for the assumption that negative parenting behaviour mediates the effects of the TASH parenting intervention. Based on these findings, a reduction in negative parenting behaviour might be an important agent of change that should be targeted by parent training. For positive parenting behaviour, we were not able to detect any mediating effect. However, given the partially inconsistent findings of this and previous studies, and the differing methods used to assess mediation, further research is required to better understand the mechanisms through which parenting interventions exert their effects. Future studies could include more mediators and consider possible moderators of the mediation process, for example age, gender, or symptom severity.
